# Mesoporous Silica Nanoparticles for Protein Protection and Delivery

**DOI:** 10.3389/fchem.2019.00290

**Published:** 2019-05-01

**Authors:** Chun Xu, Chang Lei, Chengzhong Yu

**Affiliations:** ^1^School of Dentistry, The University of Queensland, Brisbane, QLD, Australia; ^2^Australian Institute for Bioengineering and Nanotechnology, The University of Queensland, Brisbane, QLD, Australia

**Keywords:** mesoporous silica nanoparticles, mesostructure, surface modification, protein therapeutics, drug delivery

## Abstract

Therapeutic proteins are widely used in clinic for numerous therapies such as cancer therapy, immune therapy, diabetes management and infectious diseases control. The low stability and large size of proteins generally compromise their therapeutic effects. Thus, it is a big challenge to deliver active forms of proteins into targeted place in a controlled manner. Nanoparticle based delivery systems offer a promising method to address the challenges. In particular, mesoporous silica nanoparticles (MSNs) are of special interest for protein delivery due to their excellent biocompatibility, high stability, rigid framework, well-defined pore structure, easily controllable morphology and tuneable surface chemistry. Therefore, enhanced stability, improved activity, responsive release, and intracellular delivery of proteins have been achieved using MSNs as delivery vehicles. Here, we systematically review the effects of various structural parameters of MSNs on protein loading, protection, and delivery performance. We also highlight the status of the most recent progress using MSNs for intracellular delivery, extracellular delivery, antibacterial proteins delivery, enzyme mobilization, and catalysis.

## Introduction of Protein Therapeutics and MSNs

In 1922 the pancreatic insulin was successfully purified and applied for Leonard Thompson, a 14 years old boy suffering type 1 diabetes, which ushered in the era of protein therapeutics (Banting et al., 1991). Since then numerous protein drugs have been developed and used in various clinical applications. By 2008, 130 protein based therapeutics had been approved by the US Food and Drug Administration (FDA) and the number of approved protein drugs soared to 239 in 2017 (Leader et al., 2008; Usmani et al., 2017). In 2018, 7 of top 10 best-selling human drugs are proteins based ones (Urquhart, 2018). Those protein therapeutics comprise enzymes, monoclonal antibodies, vaccines, hormones, growth factors, tumor necrosis factors, etc., (Usmani et al., 2017). Protein based drugs are receiving growing interest due to their specific functions, less side effects, which are also considered safer than gene therapy as no genetic change happens (Gu et al., 2011). However, the wide applications of protein drugs are hindered due to their intrinsic drawbacks especially low stability. The folded characteristic 3 dimensional structures of proteins are essential for their biological functions, but the conformation is only slightly more stable than unfolded one. From an entropic point of view proteins are easy to be denatured (Villegas et al., 2018). In addition, some therapeutic proteins need to act inside cells, thus intracellular delivery of active forms of proteins into specific cells remains the main challenge of such proteins drugs (Ghosh et al., 2010; Gu et al., 2011).

The rapid development of nanotechnology provides a revolutionary way in the design of nanoparticle based drug delivery systems to protect proteins and deliver them to desired places. New formulations based on nanoparticles or nanostructures have already been used in the clinical setting (Peer et al., 2007; Davis et al., 2008) and have demonstrated enhanced efficacy and reduced side effects, due to the properties brought on by nanoscale effects (Muller et al., 2002; Torchilin, 2005; Naseri et al., 2015). Nowadays, the clinically available delivery systems are mainly organic materials such as liposomes and other lipid formulations and polymers (Gradishar et al., 2005; Sparreboom et al., 2005; Duncan, 2006; Greco and Vicent, 2009). However, the intrinsic instability and limited drug-loading capacity inhibit their applications for protein delivery (Elsabahy and Wooley, 2012; Chen et al., 2013).

Recently, the development of inorganic materials such as MSNs, quantum dots (Gao et al., 2004; Michalet et al., 2005), carbon-based nanomaterials (Liu et al., 2011; Robinson et al., 2011), layered double hydroxides (Bao et al., 2011; Yan et al., 2013; Kura et al., 2014) and magnetic nanoparticles (Arruebo et al., 2007; Sun et al., 2008) have attracted great attention due to their remarkably high chemical stability. Among this group of carriers, MSNs are of special interest because of their excellent biocompatibility, high drug loading capacity, rigid framework, well-defined pore structure, easily controllable morphology, and tuneable surface chemistry (Lind et al., 2003; Meng et al., 2011; Chen et al., 2013; Xu et al., 2014). The delivery of proteins using traditional MSNs is usually limited by the small pores. Recent development of MSNs with large pores and novel pore structures greatly expand their applications for protein therapeutics delivery (Shen et al., 2014; Knezevic and Durand, 2015; Xiong et al., 2015; Xu et al., 2015; Yang J. P. et al., 2015). In addition, with abundant surface modification, various responsive release systems based on MSNs have been developed with numerous advantages such as improved efficacy and reduced toxicity (Zhu et al., 2017). In this review, how to design MSNs for achieving effective protein loading, protection and delivery will be comprehensively reviewed. The progress of MSNs based protein therapy for various applications including intracellular delivery, extracellular delivery, antibacterial proteins delivery, enzyme mobilization and catalysis will be highlighted.

## Engineering MSNs for Protein Loading, Protection, and Delivery

Encapsulation of proteins within nanocarriers can overcome the shortcomings of proteins such as poor solubility, poor stability, difficulty in crossing the cell membranes and lack of specificity. In addition, nanocarriers enable the delivery of unique drug combinations which are important for personalized medicine (Mura and Couvreur, 2012; Kim et al., 2013). Compared to current clinically used organic nanocarriers such as liposomes, MSNs can achieve higher protein loading capacity due to their large pore size, high surface area and large pore volume. In addition, it is reported that the solid frame of MSNs would protect the proteins from denaturation (Kao et al., 2014). A large number of MSNs with different structures, morphology, and surface functionalization have already been designed and applied for drug delivery (Carino et al., 2007; Vallet-Regi et al., 2007; Angelos et al., 2008; Wang, 2009; Manzano and Vallet-Regi, 2010; Yang et al., 2012; Chen et al., 2013; Shen et al., 2013; Siefker et al., 2014; Dai et al., 2017). In the following part, the effects of pore size, surface functionalization, pore structure, pore volume and surface area on the protein loading and protection ability are reviewed.

### Pore Size

In order to load proteins into the mesopores, the pore sizes of MSNs usually need to be larger than the protein molecule dimensions. MSNs with larger pore sizes usually have higher drug loading amounts and faster release rates compared to the ones with small pores, which may be due to a steric hindrance effect (Vallet-Regi et al., 2008; Cirujano et al., 2017). In one study when the pore sizes of SBA-15 were varied from 8.2 to 11.4 nm, the bovine serum albumin loading ability was increased from 15 to 27% (Vallet-Regi et al., 2008). Zhang et al. (2014) prepared a series of hydrophobic silica vesicles with different entrance sizes ranging from < 3.9 to 34 nm (< 3.9, 6, 8, 13, 16, 24, 33, 34 nm) and tested the loading capacity of RNase A (with dimension of 2.2^*^2.8^*^3.8 nm). Silica vesicles with pore size of 6 nm exhibited the highest RNase loading amount (563 mg/g), which was almost double of that achieved by silica vesicles with small pores (< 3.9 nm) or large pores (>13 nm). This effect was also observed in other mesoporous structures such as MCM-48 with a 3D cubic pore structure. MCM-48 with a pore size of 5.7 nm exhibited a higher loading capacity of ibuprofen (IBU) compared to the one with 3.6 nm pores, and a faster release rate (Izquierdo-Barba et al., 2005).

**Figure 1:**
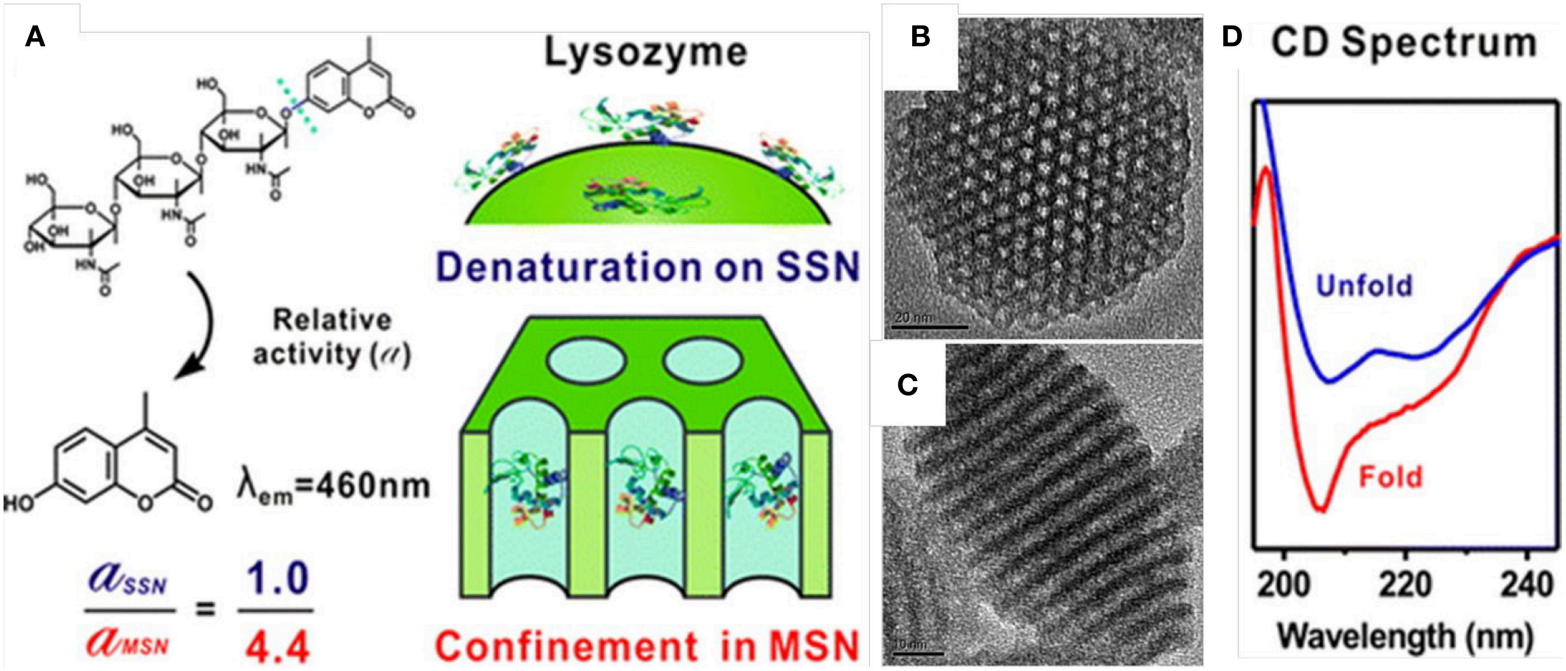
Enhanced stability and activity of lysozyme after loaded inside the mesopores of MSNs. Schematic illustration **(A)** showed the relative activity of lysozyme loaded into MSNs was 4.4-folds higher than that loaded on the outer surface of solid silica nanoparticles (SSN). **(B,C)** showed the pore structure of MSNs and **(D)** showed the circular dichroism (CD) spectrum of free lysozyme and the one loaded inside MSNs. Reproduced with permission from Kao et al. (2014), The American Chemical Society.

The enhanced activity and stability of proteins, once loaded inside the pores of MSNs, have been well-documented. Kao et al. (2014) studied the activity and stability of lysozyme immobilized in MSNs of various pore sizes by testing the proteins' secondary and tertiary structures with methods such as circular dichroism and activity assay. The activity of the lysozyme when immobilized in the pores of MSNs (pore size close to protein dimensions) was higher than that of native one. In addition, the enzymatic activity was also improved by MSNs from thermal denaturation (Figure 1, Kao et al., 2014). Kalantari also reported the immobilization of another enzyme, lipase, into MSNs with tunable pore size (from 1.6 to 13 nm). They concluded that suitable pore size (slightly larger than the size of lipase) is responsible for the loading and the performance of lipase. The MSNs with optimized pore size exhibited a high loading capacity of 711 mg g^−1^, and an 5.23 times specific activity higher than that of the native enzyme (Kalantari et al., 2017).

Since the pore size of MSNs plays a critical role for the loading and release of protein, methods to control the pore size distribution should be briefly reviewed. Traditionally two ways have been developed to expand the pore size, utilizing polymers/surfactants with longer carbon chains/co-surfactants as template or adding suitable organic agents (swelling agents) to increase the sizes of surfactant templates (Knezevic and Durand, 2015). For the first strategy, the most typical example is the synthesis of SBA-15 using amphiphilic block copolymers as templates, and the pore size can achieve up to 10 nm (Zhao et al., 1998). For the second strategy, 1,3,5-trimethylbenzene (TMB) is the most common pore-expanding agent (Huo et al., 1996; Feng et al., 2000) and the pore size of MSNs can be enlarged in a large range with addition of TMB. It is noted that excessive addition of swelling agents may result in the loss of structure (Knezevic and Durand, 2015). Very recently, MSNs with radial pore structures (Polshettiwar et al., 2010; Shen et al., 2014; Du and Qiao, 2015; Wang et al., 2019) provide another strategy in the synthesis of MSNs with large pores. The pore size can be expanded to 50 nm or even larger (Xu et al., 2015; Wang et al., 2019).

### Surface Functionalization

The loading of drug into MSNs are usually achieved by the interaction between the protein molecules and surface of pore channels through non-covalent bindings such as electrostatic interaction, hydrogen bonding, pi-pi stacking etc, (Yang et al., 2012). Chemical modification of MSNs with appropriate functional groups can provide specific interactions with proteins thus provide effective control over protein loading and release. The high density of silanol groups on the surfaces of MSNs and the large library of available organic silanes make the functionalization of MSNs quite easy through a simple post-grafting or co-condensation method (Manzano et al., 2008; Yang et al., 2008; Chang et al., 2010; Li et al., 2013; Bouchoucha et al., 2014; Jambhrunkar et al., 2014). With suitable surface functionalization, strong interaction between proteins and the pore channels by electrostatic force can be achieved, and protein loading amount can be increased while release rates are slowed. In pioneering studies, positively charged amino modified MCM-41 and SBA-15 showed a much higher loading capacity to IBU (a drug with carboxy groups, negative charged) compared to unmodified negative charged ones (Vallet-Regi, 2006). A slower release rate of IBU was also observed from the amino modified MSNs (Babonneau et al., 2003, 2004; Ramila et al., 2003; Song et al., 2005; Vallet-Regi, 2006). Tu et al. (2016) tested the encapsulation ability of negatively and positively charged MSNs with big pores (10 nm) toward a series of proteins with different molecular weights (from 12 to 250 kDa) and surface charges. It is concluded that the surface chemistry within the channels plays a dominant role in the loading of proteins. It is also notable that the protein loading process was quick, MSNs achieved 95% of maximum proteins loading ability within 20 min (Tu et al., 2016).

Another strategy of surface functionalization to control the protein loading and delivery behaviors is modification of MSNs with hydrophobic groups. Proteins are composed of many amino acids with different hydrophobic properties, a hydrophobic surface modification usually increases the protein loading and enhance the stability. Doadrio et al. (2006) modified SBA-15 with octyl (-C8) and octadecyl (-C18) groups and tested the drug release behaviors after loading with an antibiotic drug erythromycin. They found the MSNs modified with hydrophobic groups showed a slower release rate, the octadecyl-modified SBA-15 exhibited a one order of magnitude lower release rate compared to unmodified SBA-15. The observation was explained as the hydrophobic groups impeded the penetration of aqueous solution and prevented the fast release of the loaded drugs (Vallet-Regi et al., 2007). Bale et al. (2010) utilized n-octadecyltrimethoxysilane modified silica nanoparticles to deliver green fluorescent protein and RNase A into mammal cells. Results indicated that hydrophobic modification helped to preserve the biological activity of proteins and, more importantly, to achieve endosomal escape. Niu et al. (2016) studied the effects of hydrophobic modification (octadecyl-group) as well as surface roughness of silica nanoparticles on the loading capacity, release profile, cellular uptake and endosomal escape of RNase A. They concluded that the hydrophobic modification enhanced the protein loading capacity, achieved sustained release and improved the cellular uptake performance. Octadecyl-functionalized silica nanoparticles with rough surface showed the best performance in RNase A delivery which caused significant cancer cell inhibition. In addition, Zhang et al. (2018) reported that hydrophobic modification of silica vesicles (-C8 and -C18 groups) enhanced the insulin enrichment ability from PBS or artificial urine. They also found that the insulin which loaded inside alkyl modified silica vesicles showed less secondary structure's conformation change than that of hydrophilic ones.

### Pore Structure

**Figure 2:**
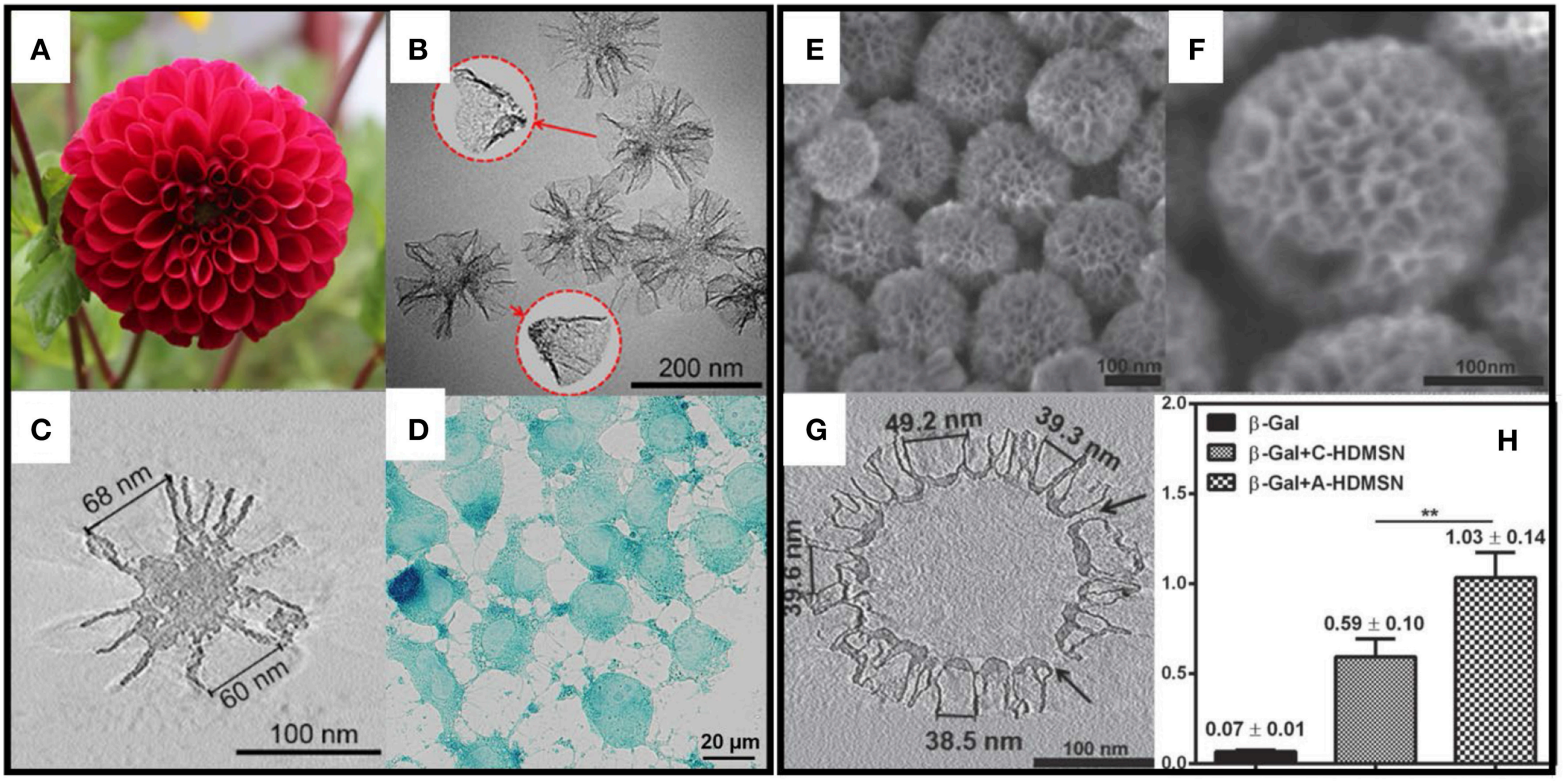
MSNs with radial pore structure and their application for large protein (β-Gal) delivery. **(A–C)** showed the structure of MSN-CC and **(D)** shows the intracellular delivery of β-Gal. **(E–G)** showed the structure of amino group modified hollow MSNs with radial pores. **(H)** showed the highest β-Gal delivery efficacy ** *p* < 0.01. Reproduced with permission from Xu et al. (2015), The Wiley-VCH and Meka et al. (2016), The Wiley-VCH.

Various pore structures, in terms of pore geometry, are also reported to affect the protein loading and release properties. Xu et al. (2015) synthesized MSNs with cone shaped pores (MSN-CC, Figures 2A–D), which has a large pore size (45 nm) and a high pore volume (2.59 cm^3^ g^−1^). They demonstrated that MSN-CC can achieve a high loading capacity of large proteins and successfully deliver active beta-galactosidase (β-Gal, 8^*^13^*^18 nm) into cells. Based on this work, Meka et al. (2016) designed an amine-functionalized hollow MSNs with cone shaped pores using one step synthesis. With the cationic groups, this hollow MSNs (Figures 2E–H) showed higher loading capacity toward negative proteins such as β-Gal and better cellular uptake performance by up to 40-fold and 5-fold compared to free protein or protein loaded in unmodified MSNs. In addition, β-Gal delivered by amine-modified MSNs retains its activity and catalytic functions. Andersson et al. (2004) also showed MSNs with cage-like pores provided a higher drug loading amount compared to those with cylindrical pores. The pore structure also influences the drug release behavior. Vallet-Regi et al. (2007) found that MCM-48 with a 3D cubic pore structure released loaded IBU faster than MCM-41 with 2D hexagonal pores (Izquierdo-Barba et al., 2009).

### Surface Area

Usually the drug loading process was carried out by immersing MSNs in drug solutions with high concentration followed with separation. Vallet-Regi et al. (2007) compared the maximum loading amount of alendronate in MSNs with similar structure but different surface area. Results showed that under the same loading condition MCM-41 with surface area of 1,157 m^2^ g^−1^ had a higher loading amount than SBA-15 with surface area of 719 m^2^ g^−1^ (139 vs. 83 mg g^−1^) (Vallet-Regi et al., 2007; Izquierdo-Barba et al., 2009). The pore surface provides the sites for the physical or chemical adsorption of the drugs, thus is an important factor for evaluating the drug loading capacity of MSNs. This conclusion is based on the studies of small molecular drugs. For proteins, large pore negative charged MSNs with different structures (with a core inside vs. hollow) but similar surface area have similar proteins loading capacity (Xu et al., 2015; Meka et al., 2016). More studies with rationale design are suggested to further test the effects the surface area on protein loading. It is noted that the contribution of different (e.g., micropore) surface area need to be considered corresponding influence on protein loading and release.

### Pore Volume

Though the drug loading process is considered to be mainly happened on the surface of mesopores, the drug-drug interactions can happen under some conditions such as very high drug loading concentration, which could fulfill the pores. In those cases the pore volume is an important factor which affects the drug loading capacity. For example mesocellular silica foams with a pore volume of 1.9 cm^3^ g^−1^ showed a higher bovine serum albumin loading amount than SBA-15 with a pore volume of 1.1 cm^3^ g^−1^ (Schmidt-Winkel et al., 1999). Yang and co-authors coated mesoporous silica foam (pore size > 10 nm) on the outside of solid magnetic oxide composites for protein adsorption. With the addition of several mesoporous silica layers, the pore volume increased to ~0.49 cm^3^ g^−1^ and high loading capacity toward BSA (113 mg g^−1^) and cytochrome C (142–175 mg g^−1^) were achieved without compromising the magnetic property (Yang et al., 2014). Xu et al. (2015) synthesized MSNs with cone shaped pores and the pore volume reached as high as 2.69 cm^3^ g^−1^, a ultra-high loading capacity toward large proteins (560 mg g^−1^ toward IgG and 190 mg g^−1^ toward β-Gal) was achieved (Xu et al., 2015; Meka et al., 2016). In general, MSNs with high pore volume can load more amount of proteins under the condition that the pore size is larger than the dimension of proteins. The effect of pore volume toward protein release has not been reported yet to our knowledge.

## Application MSNs for Intracellular Proteins Delivery

Protein therapeutics are promising drugs to intervene cell functions more precisely due to their high target specificity. They are also considered to be safer compared to gene therapies as no genetic alteration happens. In many applications such as cancer therapy and immune therapy, protein therapeutics need to work inside the cells however bare protein cannot cross the cell membranes by themselves. In 2007, Slowing et al. (2007) first demonstrated the intracellular delivery of a small protein, native cytochrome c (with a size of 2.6^*^3.2^*^3.3 nm), into human cervical cancer cells (Hela cells) by MCM-41 type MSNs with 5.4 nm pore size. In this pioneer work, though the intracellular delivery of cytochrome c was proved, the function of the protein after deliver into cells was not tested. Later, Davis et al. (2008) employed PEI modified MSNs to delivery cytochrome c and induced programmed cell death of Hela cells (Huang et al., 2013). In addition to cytochrome c, ribonuclease A (RNase A, with the size of 2.2^*^ 2.8^*^3.8 nm) is also widely used as a protein drug model to test the delivery efficacy and the intracellular functions. RNase A degraded RNA in the cytosol, after loaded into MSNs and delivered into cancer cells, they can inhabit protein production and cause cell death. Zhang et al. (2014) reported hollow silica vesicles for the intracellular delivery of RNase A. Results show a high protein loading capacity and high potency for cancer cell inhibition. Niu et al. (2016) demonstrated hydrophobic modification (C18-functionalization) of MSNs is an effective strategy for the intracellular delivery of RNase A. Benzene-bridged MSNs (with hydrophobic groups in the framework or silica) were also fabricated and applied for RNase A delivery (Yang Y. N. et al., 2015). In addition to small proteins, protein therapeutics with large molecular weight are also delivered into cells benefiting from the development of MSNs with large pores (Xu et al., 2015; Meka et al., 2016).

**Figure 3:**
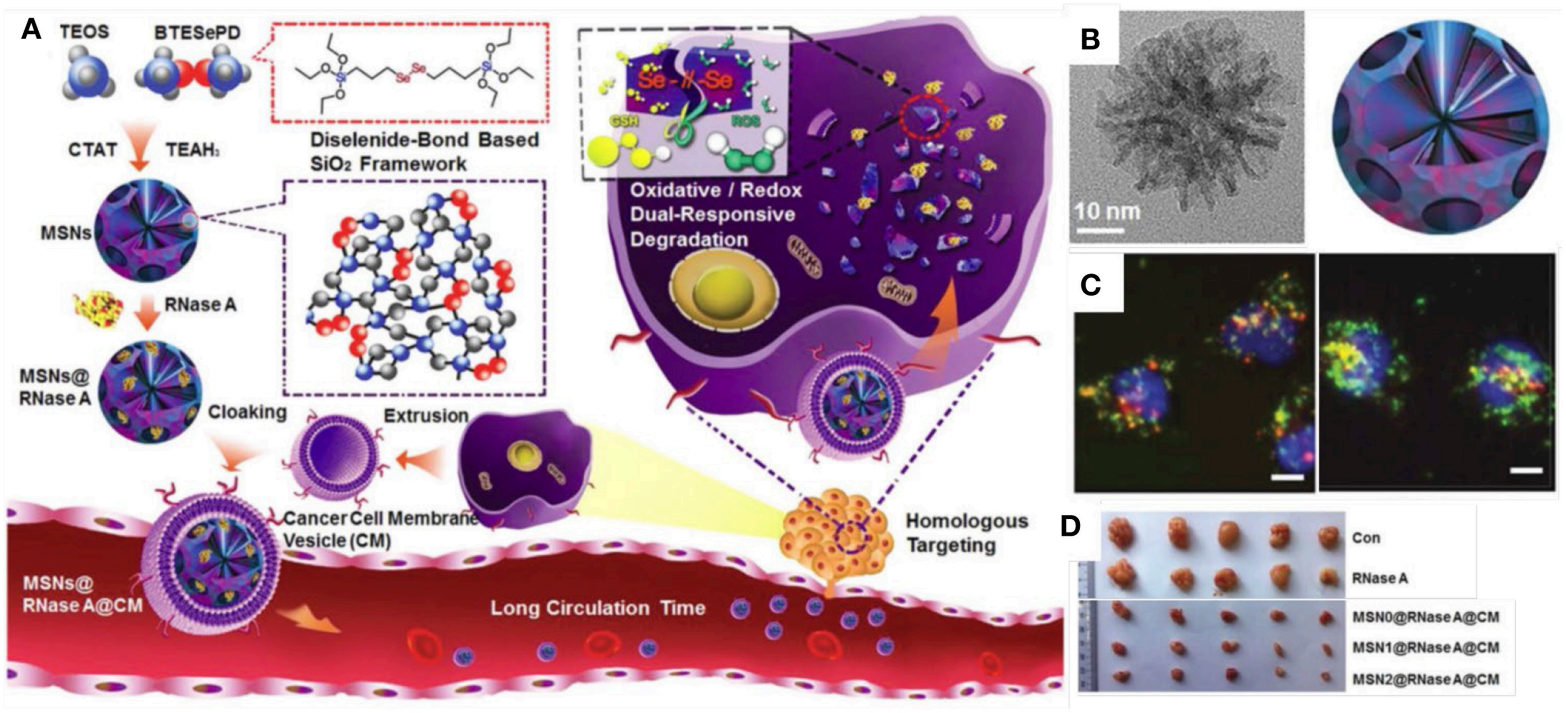
Responsive MSNs based protein delivery system for cancer therapy. Schematic drawing **(A)** showed the synthesis of biodegradable diselenide-bridged MSNs [TEM images in **(B)**] with dual-responsive and cancer cell membrane mimetic surface modification was used to deliver RNase A into cancer cells **(C)** and inhibit tumor growth *in vivo* **(D)**. Reproduced with permission from Shao et al. (2018), The Wiley-VCH.

In addition to just delivery of proteins into cells, there were more designs on MSNs to achieve “on-demand” responsive intracellular release. For example, organic MSNs with disulfide bond can achieve glutathione (GSH) responsive release to selectively release proteins in cancer cells. Yang et al. (2016) designed disulfidebond-bridged and large-pored MSNs for intracellular RNase A delivery. This disulfide bond-bridged MSNs demonstrated a GSH responsive degradation behavior, which showed a higher degradation rate in cancer cells but a low rate in normal cells. Very recently, oxidative and redox dual-responsiveness organosilica nanoparticles were further developed to selectively deliver and release RNase A in cancer cells and the anticancer performance was evaluated *in vivo* (Figure 3, Shao et al., 2018). These diselenide-bridged MSNs with 10 nm pores can load RNase A inside the pore channels with electrostatic interaction and degrade upon exposure to redox or oxidative conditions to release the payload. The anti-cancer performance was also evaluated on nude mice bearing tumors. With surface medication with fragments from the cancer cell membrane, those MSNs showed longer blood circulation time, low toxicity and enhanced tumor inhabitation ability, suggesting dual responsive degradable MSNs with proper surface modification provides a promising strategy for the delivery of protein therapeutics into tumors (Shao et al., 2018).

MSNs are also widely used for immune therapy and to deliver vaccine into antigen presenting cells (Mody et al., 2013). Yang and collaborators reported the delivery of protein antigens using multi-shell dendritic mesoporous organosilica nanoparticles for cancer immunotherapy. The organosilica nanoparticles successfully loaded ovalbumin (OVA) and mediated endo/lysosome escape to macrophages. They evaluated the *in vivo* antitumor performance of organosilica nanoparticles to deliver B16F10 tumor cell fragments in a therapeutic vaccination model, showing better immunity for cancer therapy than pure silica nanoparticles. Their work provided us new insights for the design of MSNs for adjuvants delivery and vaccine developments (Yang Y. et al., 2017). MSNs are also used for oral vaccine delivery. Wang et al. (2012) loaded bovine serum albumin into MSNs with different particle size (130 nm, 450 nm, and 1–2 μm) and administrated orally to mice. They observed the immune response and found MSNs with small size triggered higher IgG antibody concentration in plasma (Wang et al., 2012).

**Figure 4:**
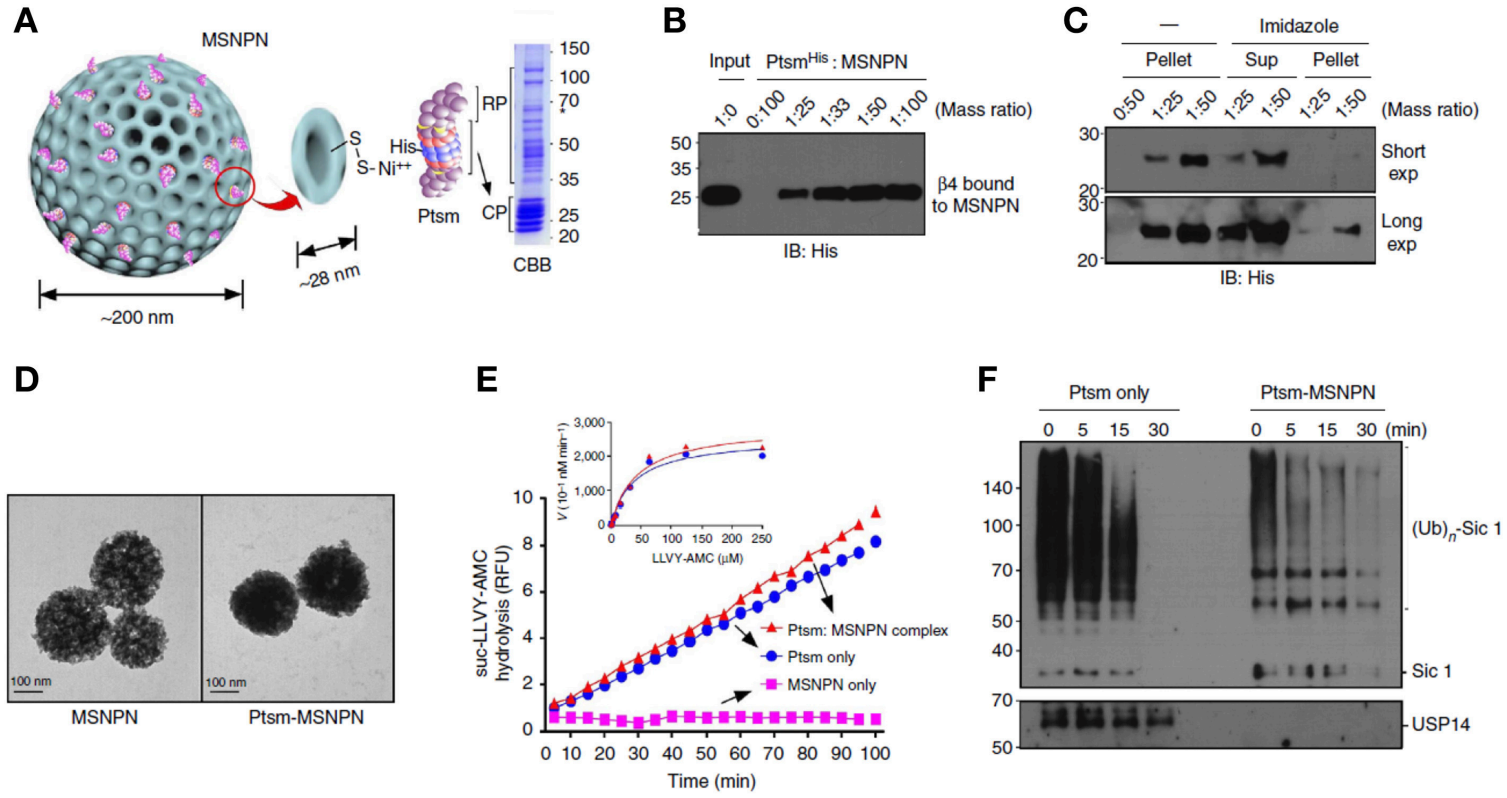
MSNs delivered proteasome to degrade tau aggregates, a pathological hallmark of Alzheimer's disease. Panel **(A)** was the schematic illustration and **(B,C)** showed the SDS-polyacrylamide gel electrophoresis (PAGE) staining of MSNs- proteasome interaction. Panel **(D)** showed the TEM images of MSNs and proteasome loaded MSNs. Hydrolysis assay **(E)** and western blots studies **(F)** demonstrated the degradation of tau aggregates, indicating the delivery of active form of proteasomes by MSNs. Reproduced with permission from Han et al. (2014), The Nature Publishing Group.

In addition to cancer and immune therapy, MSNs are also used to for other protein therapies such as deliver proteasomes for the treatment of Azhamen's syndrome. Han et al. (2014) utilized MSNs to load and deliver therapeutic proteasomes to degrade tau aggregates for the management of Alzheimer's disease. MSNs were internalized and distributed in the cytosol after endosomes escaping. *In vitro* tests showed proteasomes loaded MSNs degraded the overexpressed tau in the cells more efficiently compared to the native proteasomes, and decreased the levels of the truncated tau which is considered as pathological hallmark of this disease (Figure 4).

## Application of MSNs for Extracellular Protein Delivery

For those protein therapeutics that works outside of cells, MSNs also provide a platform to protect their activity and achieve responsive release. For example, insulin is widely used for the management of diabetes. However, the daily multiple insulin injections are quite painful, this discomfort can become a barrier to the use the insulin injections for many patients (Hunt et al., 1997; Zambanini et al., 1999). In addition, direct injection manner may cause hypoglycemia and result in serious problems such as unconsciousness or even death (Veiseh et al., 2015). Glucose responsive systems that release insulin automatically in a way that mimics physiological insulin secretion provide a better way and have the potential to change the way in which type 1 diabetes is managed.

Various MSN-based glucose responsive insulin release systems have been developed which take advantage of the high drug loading capacity, good biocompatibility and easy surface modification offered by MSNs (He and Shi, 2011; Zhao et al., 2011; Chen et al., 2013; Xu et al., 2017). In 2009, Zhao et al. (2009) reported boronic acid (one type of phenylboronic acid, PBA, which can form reversible covalent complexes with diol units of glucose) functionalized MSNs for glucose-responsive controlled release of insulin and cyclic adenosine monophosphate. The gluconic acid-modified insulin was immobilized on the exterior surface of MSNs, which also served as caps to encapsulate cAMP molecules inside the mesopores. The release of both insulin and cAMP was triggered by the introduction of glucose, which competitively bounds to boronic-acid on the surface of MSNs, resulting in the loosening of insulin and the release of cAMP. However, in this work the insulin was modified by gluconic acid which may affect the biological function of this component. Sun et al. (2013) introduced another two PBA derivatives, 3- acrylamidophenylboronic acid and N-isopropylacrylamide for use as capping agents for insulin loaded MSNs. These PBA derivatives formed a dense layer which prevented the release of insulin and underwent swelling upon exposure to glucose to trigger insulin release. In this design unmodified insulin was used which eliminated the concern of denaturation of insulin.

Another design based on GOD mechanism was reported in 2011. Zhao et al. (2011) used MSNs with large pores (approx. 20 nm) for insulin loading, while the pore capping was achieved via a coating of GOD and catalase (CAT), an enzyme capable of catalyzing H_2_O_2_ into H_2_O and oxygen to prevent the accumulation of H_2_O_2_, using layer-by-layer (LbL) method to control the insulin release. Up to 377 mg/g loading capacity of insulin was achieved using this method. The glucose responsive layers (enzyme layers) were coated onto the insulin loaded MSNs by Schiff base bond formation and functioned as “gates” to preventing insulin release in the absence of glucose. The enzymes (GOD and CAT) converted glucose into gluconic acid with oxygen and the production of gluconic acid decreased the local pH value. In the presence of glucose, the Schiff base bond was partially protonated and the enzyme layers were “loosened” which increased the permeability and triggered insulin release (Qi et al., 2009; Chen et al., 2011, 2012). With this design the insulin was released in response to glucose spontaneously and could achieve repeated on/off releases of insulin under the condition with/without glucose (Zhao et al., 2011).

**Figure 5:**
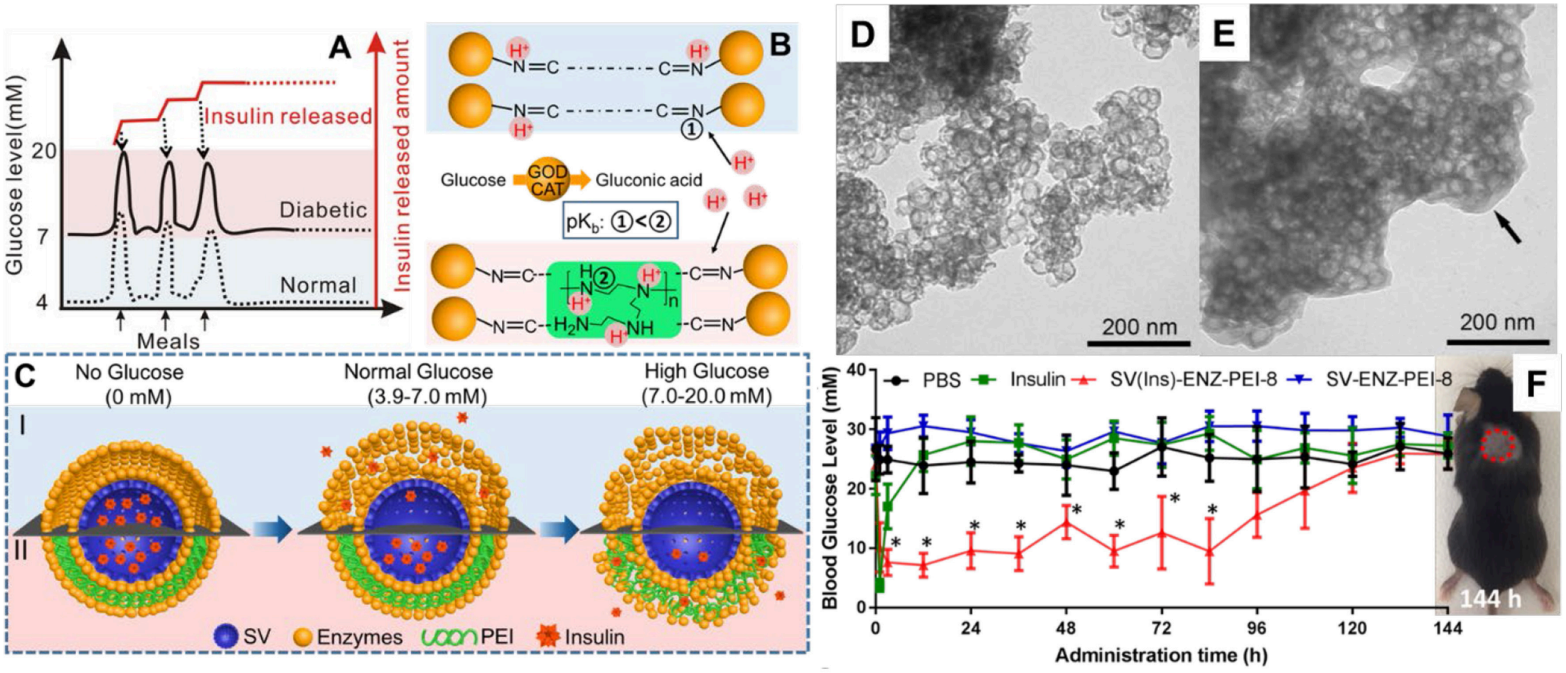
MSNs based glucose responsive insulin delivery system **(A–C)**. Hollow MSNs **(D)** was used to loaded insulin and functionalized with glucose responsive layers through enzyme-polymer layer-by-layer coating strategy **(E)**. *In vivo* studies showed MSNs based nanosystem enables a fast glucose response insulin release and regulates the glycemia levels in a normal range up to 84 h with a single administration **(F)**. Reproduced with permission from Xu et al. (2017), The American Chemical Society.

It is noted that most of current glucose responsive insulin release systems (primarily GOD based systems) release more than half their loaded insulin at a glucose concentration either below 7 mM (De Geest et al., 2006; Ding et al., 2009; Qi et al., 2009; Wang et al., 2009; Zhao et al., 2009, 2011, 2012, 2013; Chen et al., 2011, 2012; Sato et al., 2011; Sun et al., 2013; Chou et al., 2015) or above 20 mM (Gu et al., 2013; Yu et al., 2015). However, the blood glucose levels are adjusted in the range of 3.9 ~ 6.1 mM under normal physiological conditions, which means most of the glucose responsive systems are too sensitive, releasing more than half the loaded insulin content even under normal blood glucose concentrations. Recently, Xu et al. (2017) reported a glucose-responsive insulin release system based silica vesicles loaded with insulin with a layer-by-layer enzyme polymer coating (Figure 5). The insulin-release threshold can be adjusted by changing the polymer amount in the coating layers and the insulin release was switched “ON” in response to hyperglycemia and “OFF” to normal glucose levels. *In vivo* experiments in type I diabetes mice showed this MSNs based system regulated the glycemia levels in a normal range up to 84 h with a single administration while not affected the blood glucose concentration of normal mice. Those MSNs based systems have the potential to be developed as convenient and safe insulin delivery carriers for diabetes management.

For monoclonal antibodies generally working on the surface of cells, loading inside MSNs also enhanced their activity by providing protein and controlling release. For example, cytotoxic T-lymphocyte associated antigen 4 antibody (CTLA-4 Ab) can inhibit checkpoint receptor and has been used in patients with melanoma. Functionalized silica foam with a pore size of 30 nm was used to loaded CTLA-4 Ab and showed an ultra-high loading capacity (up to 800 mg g^−1^). *In vivo* tests with tumor bearing mice (melanomas) model showed that CTLA-4 Ab loaded silica foam significantly enhanced antitumor activity compared to free antibodies, attributed to the prolonged release and protection of antibodies at tumor sites (Lei et al., 2010).

## Application of MSNs for Antibacterial Proteins Delivery

**Figure 6:**
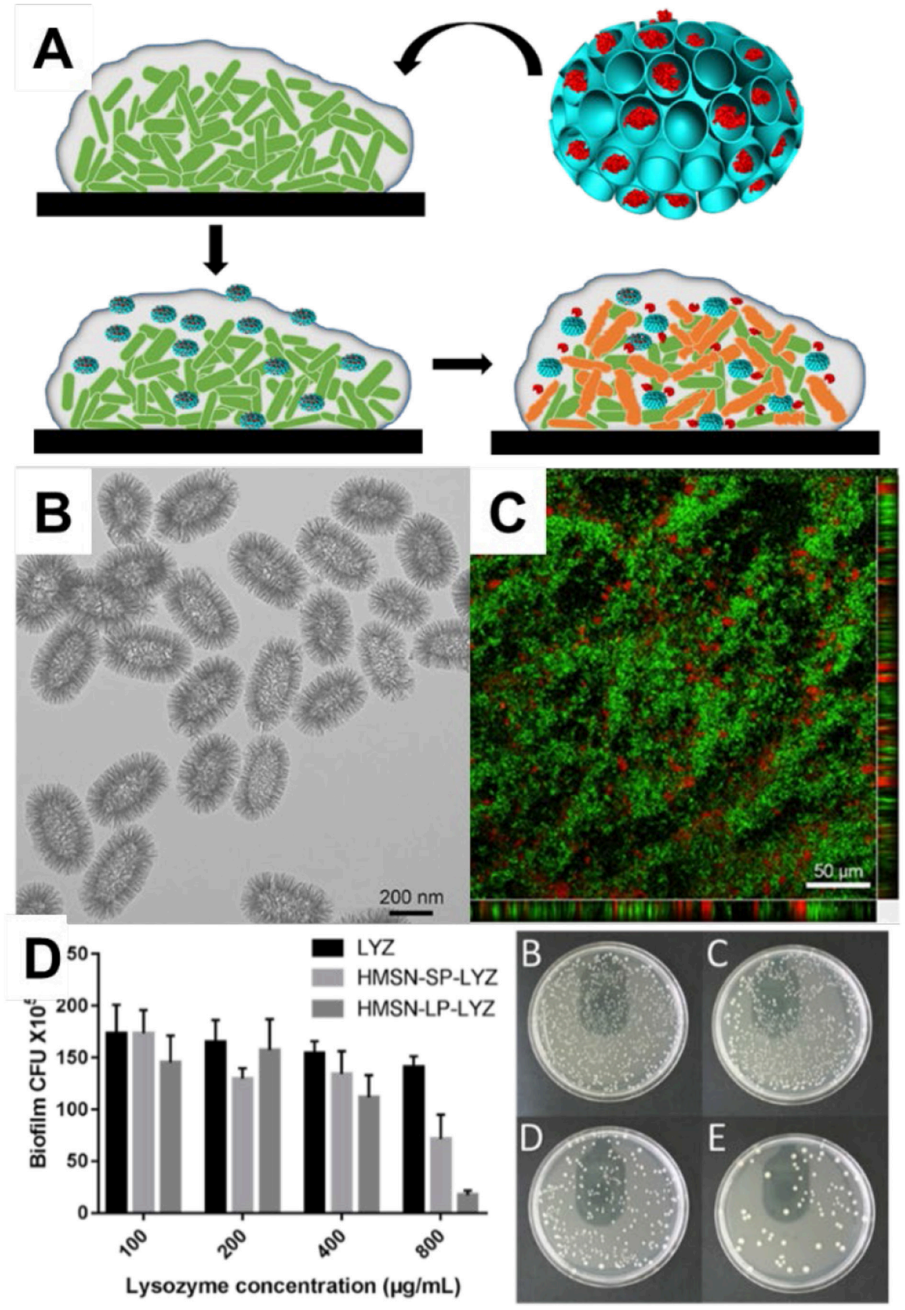
Mesoporous silica nanoparticles for the delivery of antimicrobial protein into biofilm. MSNs for lysosome delivery. **(A)** the schematic drawing of MSNs delivery for biofilm. Panel **(B)** showed the TEM image of MSNs and **(C)** the penetration of MSNs into biofilm. The antibacterial performance was tested towards *E. coli* biofilm **(D)**. Reproduced from Xu et al. (2018) and by permission of The Royal Society of Chemistry.

The use of nanoparticles as delivery vehicles for antimicrobial proteins shows great potential for the treatment of bacterial infections. For example, lysozyme, a nature protein than can catalyze the hydrolysis of bacterial wall, was coated on the surface of MSN-41 which enhanced the interact with *Escherichia coli* (*E. coli*, one typical Gram-negative bacterium) and raised the local concentrations of lysozyme. The minimal inhibition concentration was 5-folds lower after conjugated with MSNs compared to free lysozyme (Li and Wang, 2013). To tackle the problem of exposure of lysozyme on the external surface, Song et al. (2016) prepared MSNs with large pores which had ability to load lysozyme inside, and demonstrated the enhanced the ability for the treatment of *E. coli in vitro* and in an *ex vivo* small intestine infection model. Wang et al. (2019) prepared dendritic mesoporous silica nanoparticles with pore sizes ranging from 2.7 to 22.4 nm for lysozyme loading. They found MSNs with large pores had a high lysozyme loading ability (244.5 mg g^−1^) and showed a sustained release profile. Lysozyme loaded inside MSNs showed better antibacterial effect toward *E. coli*, reducing the minimum inhibitory concentration (MIC) from 2,500 mg mL^−1^ of free lysozyme to 500 μg mL^−1^. Very recently, Xu et al. (2018) reported that MSNs could penetrate inside the biofilms (Biofilms are groups of microbial cells embedded in extracellular polymeric substances and bacteria in biofilms had higher resistance to antimicrobial drugs) and deliver lysozyme into biofilm to kill deeper bacteria (Figure 6A). Those hollow mesoporous silica nanoparticles with large cone-shaped pores (Figure 6B) had ability to loaded lysozyme inside and penetrated into biofilms (Figure 6C). Enhanced therapeutic activity toward E. coli biofilms was demonstrated with rational design of MSNs (Figure 6D).

## Application of MSNs for Enzyme Mobilization and Catalysis

MSNs are also of great significance for enzyme immobilization and catalysis by addressing the intrinsic issues of the native enzymes (Wang and Caruso, 2005; Popat et al., 2011; Yang T. et al., 2017). Wang and Caruso (2005) used a series of MSNs with pore sizes from 2 to 40 nm for the immobilization of various enzymes including lysozyme, peroxidase, catalase and cytochrome C. After loading inside MSNs, the enzymatic activity was retained in a wide range of pH and even after exposure to enzyme-degrading substances such as proteases. It is noted that MSNs-enzyme kept 70% of the initial activity after 25 batch of successive reactions. Very recently, Kalantari et al. (2018) also reported the application of dendritic mesoporous organosilica nanoparticles with benzene groups in the framework for an enzyme, lipase, and immobilization. It is interesting to note that after loaded into organosilica nanoparticles, lipase showed enhanced pH and thermal stability and also higher activity than free lipase. In addition, after 5 cycles lipase loaded in MSNs retained 94% catalytic activity, showing the advantage for reusability (Kalantari et al., 2018).

## Summary and Outlook

In conclusion, MSNs demonstrated high loading capacity and protective effects toward proteins, provided advantages in the intracellular, extracellular, antibacterial delivery, immobilization of various proteins with enhanced therapeutic/catalytic efficacy. With the rigid framework and well-defined pores, MSNs provide protection toward protein and preserve their activity. In addition, the fast development of novel MSNs especially those with radial pore structure and large pores promotes the application for protein delivery. We envision that significant progress will be made and new MSNs with rational design and tailored functionalization will be developed in the near future for better protein delivery.

For the future directions, targeted protein delivery and controlled protein release would be emerging technological strategies to further improve the therapeutic effects. The recent works such as cloaked MSNs with red blood cell membranes or other targeting agents have shown longer circulation time and accumulation in target areas such as tumor (Xuan et al., 2018). The design of various responsive release system based MSNs are also receiving more attention. Many new studies have clearly demonstrated the feasibility and advantage of remote-controlled proteins release systems (Yang et al., 2013).

It is noted that the *in vivo* effects of MSNs based proteins delivery systems are less studied. More intensive preclinical explorations such as animal studies are needed to realize their potential in clinical applications. Currently the investigation of MSNs for the *in vivo* delivery of therapeutic proteins has not kept pace with advances in MSNs fabrication. More studies are expected to evaluated the biocompatibility, stability, efficacy and biological interactions of MSNs based protein delivery system. The close collaborations between materials scientists, biologist, pharmacist, and clinician would fasten this process.

## Author Contributions

CX, CL, and CY designed this study. CX and CL wrote the manuscript. CX and CY revised the manuscript.

### Conflict of Interest Statement

The authors declare that the research was conducted in the absence of any commercial or financial relationships that could be construed as a potential conflict of interest.
